# Post-trauma complex orthodontic approach: the impact of psychological issues of bullying on treatment decision

**DOI:** 10.1590/2177-6709.27.4.e22bbo4

**Published:** 2022-09-23

**Authors:** Daniela FEU, Felipe de Assis Ribeiro CARVALHO

**Affiliations:** 1 Universidade Vila Velha, Departamento de Odontologia (Vila Velha/ES, Brazil).; 2 Universidade do Estado do Rio de Janeiro, Departamento de Ortodontia (Rio de Janeiro/ RJ, Brazil).

**Keywords:** Multiple impactions, Ankylosis, Dentoalveolar trauma, Bullying, Orthodontic tooth movement

## Abstract

**Objective:**

This article aims to discuss the multidisciplinary approach required in the treatment of cases of impaction and ankylosis of permanent teeth, associated with a history of trauma, considering the psychological state of the child and family when faced with a traumatic case of bullying, by reporting the complex treatment of a central incisor needing to be orthodontically moved across the midline.

**Conclusion:**

This clinical case was a major challenge, which included complex multidisciplinary procedures. Results and stability after 26 months of retention indicated successful orthodontic space closure of two maxillary teeth, without the use of implants or prostheses, in an adolescent patient who had a history of dental trauma, alveolar bone loss, and an uncertain initial prognosis.

## RESUMO

**Objetivo:**

O presente artigo tem como objetivo discutir a abordagem multidisciplinar necessária no tratamento de casos de impacção e anquilose de dentes permanentes, associados a histórico de trauma, considerando o estado psicológico da criança e da família diante de situações traumáticas de *bullying*, por meio do relato do tratamento complexo de um incisivo central que precisava ser movido ortodonticamente através da linha média.

**Conclusão:**

Esse caso foi um grande desafio, que incluiu procedimentos multidisciplinares complexos. Os resultados e a estabilidade após 26 meses de contenção indicaram fechamento ortodôntico bem-sucedido do espaço de dois dentes superiores, sem o uso de implantes ou próteses, em uma paciente adolescente que apresentava histórico de trauma dentário, perda de osso alveolar e prognóstico inicial incerto.

## INTRODUCTION

The deciduous incisors are the teeth most commonly affected by dental trauma.^1^ There is a close anatomic relationship between the apexes of deciduous incisors and the buds of succeeding teeth: the hard tissue barrier between them has a thickness of less than 3 mm and it might consist of only connective fibrous tissue.^2^ Frequent consequences for the permanent dentition after an intrusion trauma to the deciduous dentition are impaction of succeeding teeth, which can be explained by the physical displacement of the permanent tooth bud, with or without dilaceration at the time of injury; root resorption disorders^1^ ^,^ ^3^ ^,^ ^4^ and ankylosis.^5^

Impacted teeth have the potential to cause serious problems such as development of pathologies and other complications, due to their proximity to the anatomical structures.^5^ Impacted teeth can induce resorption of adjacent teeth, periodontal disease, marginal bone loss at the root surface of adjacent teeth, and cysts or tumors.^5^ ^,^ ^6^ In addition, the impaction of permanent maxillary incisors may impair the physical, psychological and social development of the child,^7^ since these teeth are in frontal and centered position in the oral cavity, playing an essential role in facial aesthetics and oral function.^8^ The treatment options for impacted maxillary incisors usually include space creation for spontaneous eruption,^9^ surgical exposure and orthodontic traction^7^, or extraction of the impacted incisor followed by prosthodontic rehabilitation.^10^ Treatment by orthodontic traction of impacted central incisors has been reported to have the most favorable outcomes, both esthetically and functionally.^11^ However, there is a risk of traction failure and bone loss.^10^ ^,^ ^12^ ^,^ ^13^

Besides, many authors state that after diagnosis of impaction, the therapeutic decision should prioritize tooth eruption.^3^ ^,^ ^7^ ^,^ ^8^ ^,^ ^11^ ^,^ ^14^ ^-^ ^17^ But it is important to emphasize that there is no standard protocol for the assessment of impacted teeth, and the decision should be taken according to each situation.^5^ In addition, the child may be the target of bullying at school, generating a negative impact on their emotional development and requiring a solution that meets the demands generated by this situation.

It is also worth mentioning that multiple dental impactions involving maxillary incisors and canines cause aesthetic, functional, psychosocial and financial burdens for affected individuals. They can cause irreparable dental harm during or after treatment, and even years after the trauma, due to sequelae.^18^ ^-^ ^24^ Considering all these points, it is crucial to intercept complex cases of impaction as soon as possible, to establish the proper therapy. Thus, this article aims to discuss the multidisciplinary approach needed in the treatment of cases of impaction and ankylosis of permanent teeth associated with a history of trauma.

## DIAGNOSIS AND ETIOLOGY

A 13-year-old girl of African descent presented to the private orthodontic office for orthodontic treatment eight years after suffering a facial trauma with traumatic dental injury (TDI), with the main complaint of *“missing front tooth and ugly smile”*. This trauma induced the avulsion of deciduous maxillary right central incisor and left lateral incisor, and intrusion of the deciduous maxillary left central incisor. After that, she attended regular preventive visits with the pediatric dentist, who detected several eruption disorders in the permanent dentition, including impaction of the maxillary left central incisor and both right and left canines. The pediatric dentist tried to use a removable appliance to move the maxillary left lateral incisor for almost a year without success, and suggested that it should be clinically diagnosed with ankylosis, due to the impossibility of induced movement.

**Figure 1:**
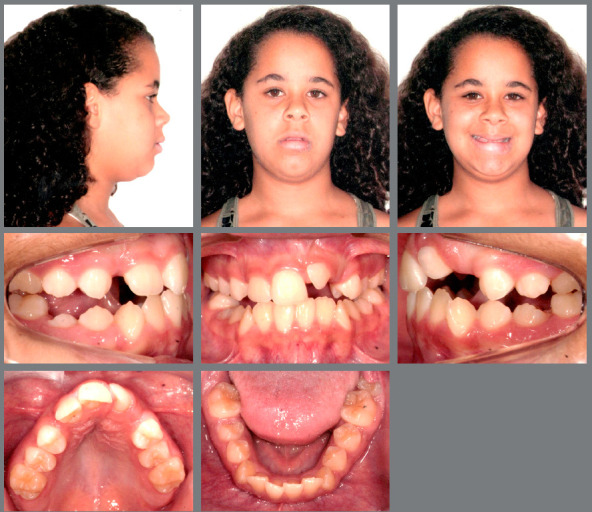
Pretreatment facial and intraoral photographs.

The patient had vertical facial pattern with a convex profile, absence of passive lip sealing and an asymmetric smile, with minimal exposure of maxillary teeth, and evidence of anterior projection of the tongue at rest and during swallowing (Fig 1). Intraoral clinical examination revealed an altered sequence of the permanent teeth eruption. There was significant positive discrepancy in the maxillary arch due to missing teeth (+9.7mm), and moderate dental crowding in the mandibular arch (-7.2mm). Occlusal analysis revealed a Class II molar relationship, with anterior and posterior open bite, reduced overjet, significant shift of the upper midline to the left side, and maxillary transverse deficiency (Fig 1). The patient had satisfactory oral hygiene, with unaltered gingiva and mucosa.

**Figure 2:**
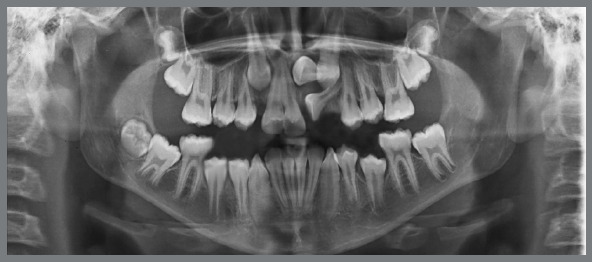
Pretreatment panoramic radiograph.

Panoramic radiograph showed the complete set of permanent teeth in different stages of formation, except for the mandibular left third molar. The maxillary right and left canines and the maxillary left central incisor were impacted. The maxillary left central incisor presented severe displacement, with an angulation greater than 90º in relation to the sagittal plane (Fig 2).

**Figure 3:**
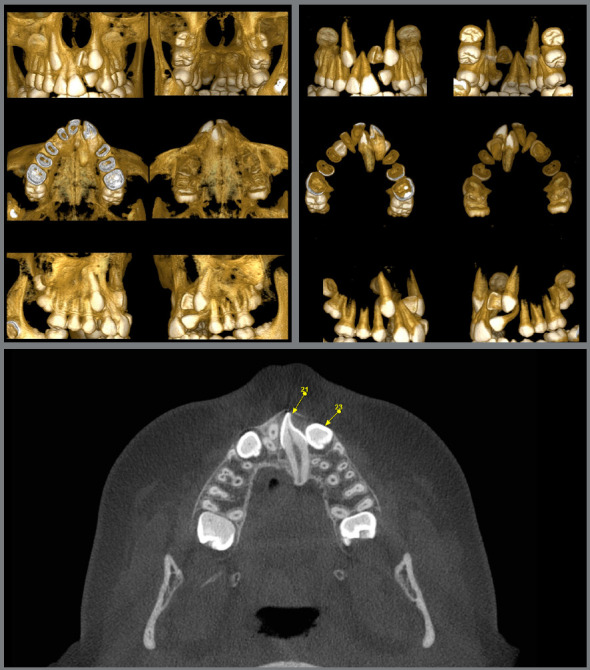
Pretreatment tomographic images.

Cone beam tomographic images (CBCT) evaluation defined the exact place of the impacted maxillary left central incisor: the tooth was horizontally positioned, and its crown was close to the nasal floor and anterior nasal spine, across the midline, while the root was palatally displaced. The distance from the cementoenamel junction to the root apex of this tooth was also 5.6mm shorter than that of the maxillary right central incisor, characterizing root shortening. The maxillary left canine root was in close relationship with the maxillary sinus (Fig 3).

**Figure 4:**
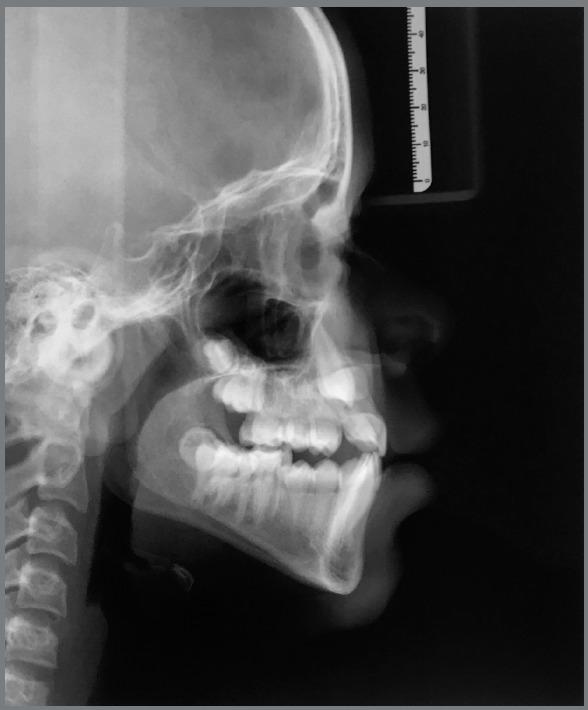
Pretreatment lateral cephalometric radiograph.

Cephalometric analysis revealed skeletal Class II malocclusion (ANB = 4.2° and Wits = 3mm) and dolichofacial pattern (SN.GoGn = 39°, FMA = 27.5°). The maxillary and mandibular central incisors were lingually tipped (U1.NA = 18°, U1-NA = 2mm, L1.NB = 19°, L1-NB = 4mm, IMPA = 83.5°) (Fig 4).

## TREATMENT OBJECTIVES

The treatment goals included: (1) expansion of the maxillary arch, recovering its ideal shape and creating space for the maxillary canines; (2) surgical exposure and forced eruption of the maxillary canines, bringing them into proper alignment, together with all their supporting tissues, thereby correcting the loss of alveolar height mainly at the left maxillary anterior region; (3) correct the habit of anterior posture of the tongue at rest and tongue-thrust swallowing; (4) obtain adequate overjet and overbite; (5) maintain the Class II molar relationship and obtain a Class I canine relationship; (6) solve the mandibular crowding; (7) establish a stable and functional occlusion; (8) increase exposure of maxillary anterior teeth during speech and smiling, providing smile aesthetics and; (9) obtain a harmonious facial profile, improving lips positioning and lip muscles tone.

## TREATMENT PLAN

The treatment plan included the use of: high-pull headgear to correct Class II and help with anchorage; Hyrax-type palatal expander, to transversely expand the maxillary arch; and extraction of the maxillary left central incisor, which had a very poor prognosis for orthodontic traction, and the maxillary left lateral incisor, with probable ankylosis. A fixed appliance was planned, with 0.022 x 0.028-in brackets (MBT prescription) in both dental arches, associated with intramaxillary and intermaxillary elastics, to reposition the maxillary anterior teeth, according to the scheme described bellow:

» Left side: replacement of the maxillary left central incisor with the maxillary right central incisor, the maxillary left lateral incisor with the maxillary left canine, the maxillary left canine with the maxillary left first premolar, and closure of any remaining space.
» Right side: replacement of the maxillary right central incisor with the maxillary right lateral incisor, the maxillary right lateral incisor with the maxillary right canine, the maxillary right canine with the maxillary right first premolar, and closure of any remaining space.

After the end of orthodontic treatment, the patient would be referred for aesthetic rehabilitation. This type of approach is rarely indicated, but it was considered the best solution for the presented situation, with lower risks and better prognosis, to meet the psychosocial, financial and aesthetic needs of the patient.

## OTHER TREATMENT OPTIONS

Certainly, there are different approaches that could be used in this complex clinical case, based mainly on the perception and experience of the clinician. The following options were also considered for this patient:

1) Extraction of both maxillary first premolars was considered for Class II correction, which would allow retraction of the maxillary canines into a Class I relationship. Opening the space for an implant at the end of treatment in the region of the maxillary left central and lateral incisors would be included in this option.
2) Another option was to insert miniplates in the maxillary bone to perform distalization of the maxillary posterior teeth, promoting the correction of the sagittal discrepancy and the opening of space in the area of the left maxillary central and lateral incisors, for the installation of implants. However, due to the young age of the patient, options 1 and 2 were not considered, due to the long waiting period until implant placement (growth completed) and/or subsequent restoration work. They were also considered impractical due to the chances of additional alveolar bone loss at the extraction site, as this region already had significant bone deficiency. In addition, the patient’s financial and psychological conditions also did not favor these two options.
3) Bilateral first premolar extractions were also considered, along with transplantation of a third molar to the area of the central incisor.^21^ This option was disregarded due to the high risk of failure associated with dental transplantation, the complexity of the procedure, considering the presence of a possibly ankylosed tooth, and the excessive costs that the patient could not afford.
4) Forcing the eruption of the maxillary left central incisor was considered at the first moment. However, the prognosis was poor, due to the fact that it was in Nolla’s stage 10, positioned inverted, and considering the root length and the risk of alveolar bone loss on the labial side^20^. Besides that, there was a great proximity between the crown of this tooth and the follicle of the maxillary left canine, which would increase the risk of compromising both dental elements. The uncertain prognosis for each tooth involved, the complexity of the movement and the possible increase in treatment time were considered in the family’s decision to extract the incisors.

## TREATMENT PROGRESS

**Figure 5:**
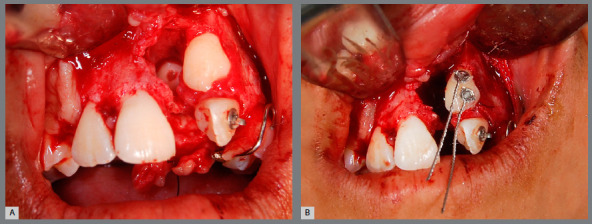
Surgical access for extraction of the maxillary left central incisor **(**A), and bonding of an orthodontic button with ligature wire extension in the retained maxillary left canine (B).

The surgical procedure consisted of a full-thickness mucoperiosteal flap to gain access to the impacted maxillary left central incisor, lateral incisor, and canine. After the osteotomy, dental section of the maxillary left central incisor and extraction (Fig. 5) was performed. During the surgical procedure, after attempts to displace the maxillary left lateral incisor, there was great difficulty, with significant mobility being observed in the region of the maxillary left canine. It was then considered that immediate extraction of the maxillary left lateral incisor would result in extensive bone loss and could compromise the stability of the maxillary left canine. So at that time, that tooth was kept. An accessory button with ligature wire extension was placed on the buccal surface of the maxillary left canine, as well as on the maxillary left lateral incisor (Fig. 5). Even though the maxillary left lateral incisor was not extracted, a surgically induced tooth dislocation was forced and then an attempt to move that tooth was initiated.

Rapid maxillary expansion was performed with a Hyrax palatal expander, activated twice daily for 28 days. After 11 mm of expansion, the Hyrax was stabilized and maintained for retention and anchorage during maxillary canine traction. Due to failure to induce movement, even after transoperative dislodgement; and a significant increase in the metallic sound to percussion^25^, ankylosis was confirmed, and the maxillary left lateral incisor was extracted. After a reasonable recovery of the bone tissue in the region of the extraction of the maxillary left central incisor, the traction of the maxillary left canine was started, by means of an elastomeric chain connecting the ligature wire to the expander bar, changed monthly until the tooth erupted in the oral cavity. 

Four weeks after the Hyrax stabilization, MBT 0.022 x 0.028-in preadjusted brackets were bonded in the maxillary and mandibular dental arches, and the high-pull headgear was adapted to Hyrax appliance bands on the first molars, with 200-g force per side, and the patient was instructed to use it at least 15 hours per day, for vertical anchorage. The maxillary right canine spontaneously erupted four months after the Hyrax stabilization. 

After nine months, the maxillary left canine erupted into the oral cavity, the Hyrax and the high-pull headgear were removed, and a bracket was bonded. Alignment and leveling were performed using 0.016-in to 0.019 x 0.025-in nickel-titanium (NiTi) heat-activated archwires. Subsequently, 0.019 x 0.025-in stainless steel archwires were placed in both dental arches to improve leveling and to close the spaces with elastomeric chain and diagonal intraoral elastics in the anterior region, to direct the midline. To maintain anchorage on the left side, Class II intermaxillary elastics were also worn for at least 15 hours a day. During treatment, compensatory torque bends were incorporated in the maxillary canines and maxillary first premolars, so that the gingival margins and the bone contour would become more esthetically adequate in their new positions after treatment completion.

In the mandibular arch, the mandibular incisor intrusion was obtained using Burstone’s three-piece intrusion arch mechanics,^26^ allowing projection of the mandibular incisors. After seven months of intrusion, the curve of Spee was corrected and continuous alignment and leveling of the mandibular arch was performed. Cautious interproximal enamel reduction of 0.3 mm was performed on the mandibular teeth to allow correction of anterior crowding and space for extrusion and alignment of the right second premolar. Alignment and leveling were performed using 0.016-in, 0.017 x 0.025-in and 0.019 x 0.025-in NiTi archwires. Subsequently, a 0.019 x 0.025-in stainless steel archwire was placed to assist in anchoring intermaxillary elastics during space closure in the maxillary arch.

**Figure 6:**
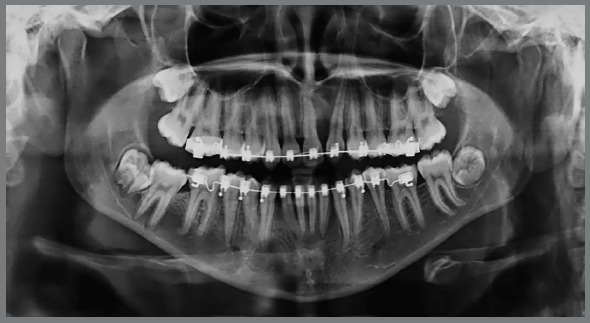
Panoramic radiograph during treatment progression, showing lack of space for eruption of the mandibular right second molar.

It was necessary to perform 0.4-mm interproximal enamel reduction between the mandibular first and second molars to allow the eruption of the mandibular second molar, which was retained due to lack of space, associated to the use of superimposed 0.016-in and 0.017 x 0.025-in NiTi archwires (Fig 6). 

Ideal occlusion was obtained and, after removal of the appliance, retention was provided by maxillary and mandibular lingual bonded bars.

The patient was referred for third molars extraction and for periodontal treatment of the maxillary left canine until periodontal and gingival health was established. A bone graft had been planned in the region of the maxillary left canine to recover periodontal support, which was not necessary, because after seven months of follow-up, with hygiene instructions and basic periodontal therapy, periodontal health was reestablished.

During the entire treatment process, the patient received speech therapy to correct the atypical positioning of the tongue, thus establishing a suitable environment for the closure of the anterior open bite, increasing the stability of the results. The position and tone of the lips were improved with the eruption of the maxillary anterior teeth and the establishment of a ideal occlusion, which allowed better working conditions for the speech therapist.

## TREATMENT RESULTS

**Figure 7:**
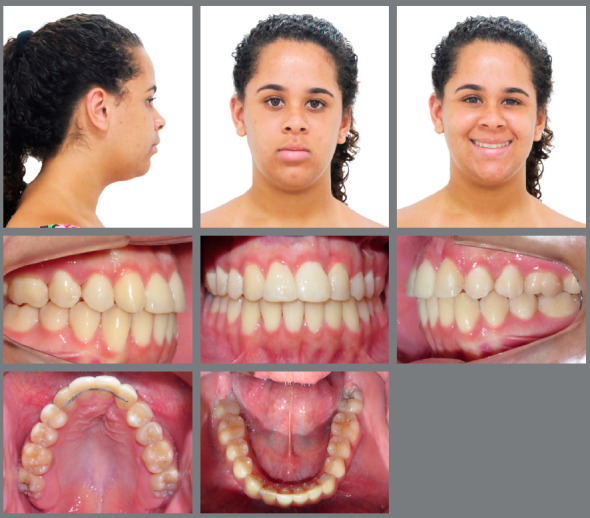
Post-treatment facial and intraoral photographs.

After 35 months, the treatment goals were achieved, with improved facial profile, symmetrical smile, good exposure of the incisors in speech and smile, and better support of the upper lip, despite a 2-mm deviation of the upper midline to the right (Fig. 7). The intraoral photos showed that bilateral Class II molar relationship was maintained, as planned, and Class I canine relationship was established. The impacted maxillary canines were aligned, with normal overjet and overbite, which facilitated restoration with composite resin buildups. The alveolar bone in the maxillary anterior region was improved, the curve of Spee was corrected as well as crowding in the mandibular arch, with excellent final intercuspation. Gingival recontouring of the right lateral incisor was performed to create an esthetically more acceptable result.^22^

**Figure 8:**
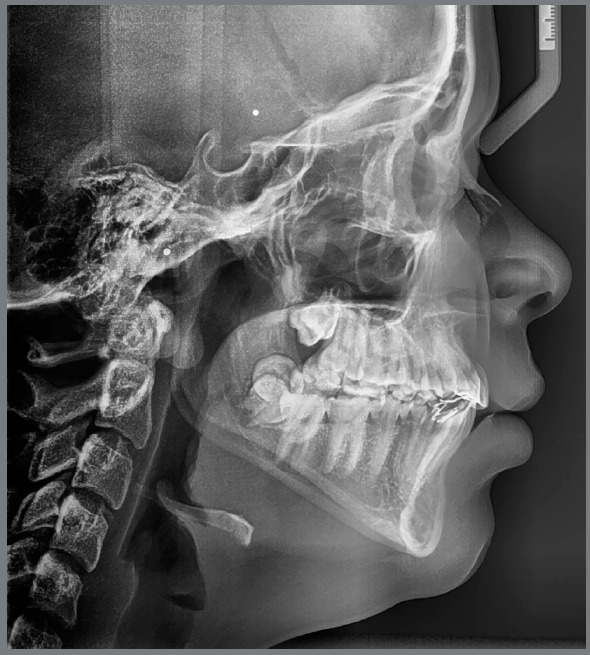
Post-treatment lateral cephalometric radiograph.

The cephalometric analysis (Fig 8, Tab 1) revealed that there were no significant changes in the position of the maxilla (SNA from 86° to 85°) and mandible (SNB from 82° to 83°), and the vertical angles were reduced (SN.GoGn from 39° to 36.5°, Y-axis from 61.5° to 58°), except for the FMA, which increased (FMA from 27.5° to 30°). Projection of the maxillary anterior teeth was observed (U1.NA from 18° to 28°; U1-NA from 2mm to 5mm) and, as expected, the mandibular incisors were also projected (L1.NB from 19° to 30.5°; L1-NB from 4mm to 6 mm; IMPA from 83.5° to 91°). 

**Table 1:** Cephalometric measurements.

MEASURES	Norm values	Pretreatment (July 2015)	Post-treatment (June 2018)	26-month retention (August 2020)
SNA (degrees)	82 ± 2	86.4	85.0	85.5
SNB (degrees)	80 ± 2	82.2	83.1	83.3
ANB (degrees)	2 ± 2	4.2	1.9	2.2
U1.NA (degrees)	22 ± 2	18.2	29.1	30.0
U1-NA (mm)	4	1.9	4.7	4.8
L1.NB (degrees)	25 ± 2	19.1	30.5	30.6
L1-NB (mm)	4	3.8	6.2	6.1
IMPA (degrees)	90 ± 2	83.5	91.4	91.8
U1/L1 (degrees)	135 ± 2	136.5	116.2	115.6
FMA (degrees)	25 ± 3	27.5	29.4	29.5
SN.GoGn (degrees)	32 ± 2	38.7	36.6	36.6
Upper Lip - Line S (mm)	0	+4	+3	+3
Lower Lip - Line S (mm)	0	+3	+3	+3

**Figure 9:**
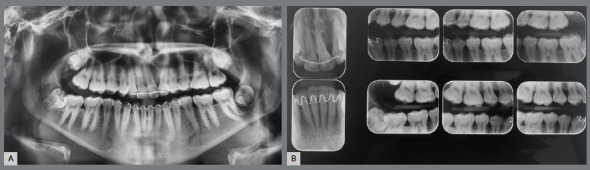
Final panoramic radiograph **(**A) and final periapical radiographs **(**B).

The panoramic radiograph (Fig. 9) showed good root parallelism, except for the maxillary left canine and the right central incisor, and no root resorption was observed. Periapical radiographs confirmed the presence of bone between the maxillary central incisor and the left canine. Periodontal probing showed no bone loss in this area, but due to the significant loss of alveolar bone height in the region at the beginning of treatment and root tipping at the end of treatment, the gingival contour was not satisfactory, and hygiene guidelines were emphasized in that region. In the other regions, periodontal conditions were good, with symmetrical and normal gingival margins, normal bone crest height and intact lamina dura, periodontal ligament and trabecular bone in the periapical area with normal levels on probing. The patient reported excellent dental function, absence of muscle pain or joint problems, satisfaction with dental and facial aesthetics, and improved quality of life.

**Figure 10:**
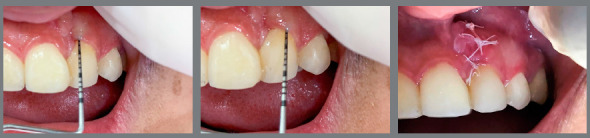
Periodontal probing and bone graft surgery in the maxillary left canine region after 18-month retention.

Periodontal probing showed no bone loss on any root surface of the maxillary left canine after 18 months of retention, and therefore, gingival graft surgery was performed to improve the contour and gingival margin of the region where the maxillary left canine was moved to (Fig 10).

**Figure 11:**
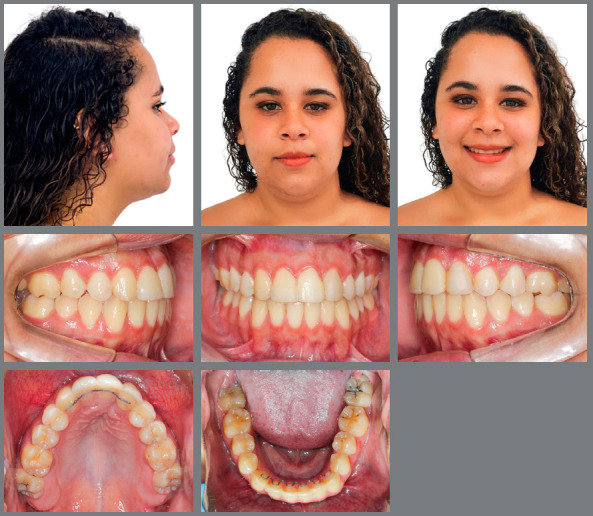
26-month retention facial and intraoral photographs.

**Figure 12:**
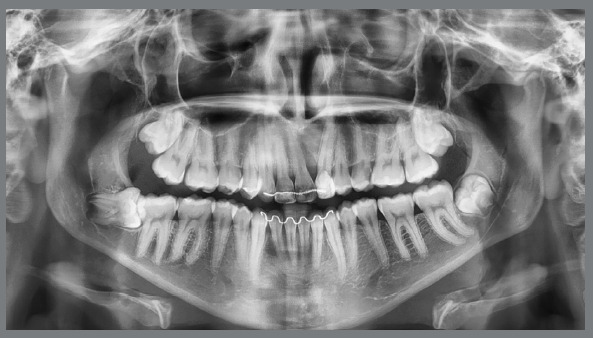
26-month retention panoramic radiograph.

**Figure 13:**
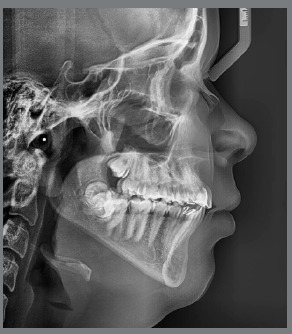
26-month retention cephalometric radiograph.

After a 26-month retention period, the facial aesthetics and occlusion achieved were maintained, and a good improvement in periodontal support and clinical health was also observed in the maxillary left canine (Fig 11). Panoramic (Fig 12) and cephalometric (Fig 13, Tab 1) radiographs did not reveal significant changes, in comparison to the end of treatment.

**Figure 14:**
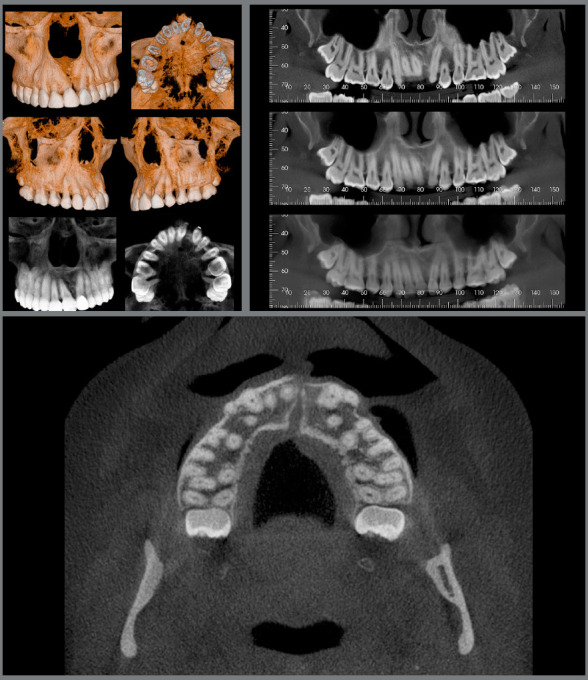
26-month retention tomographic images.

Based on the CBCT, the midpalatal suture moved together with the right central incisor, and the connective tissue of the suture was pushed to the left, apparently becoming incorporated into the periodontal ligament, appearing as a radiolucent image distal to the maxillary right central incisor (Fig 14). CBCT also revealed that the tooth movement had little or no effect on the position of the incisive foramen.

**Figure 15:**
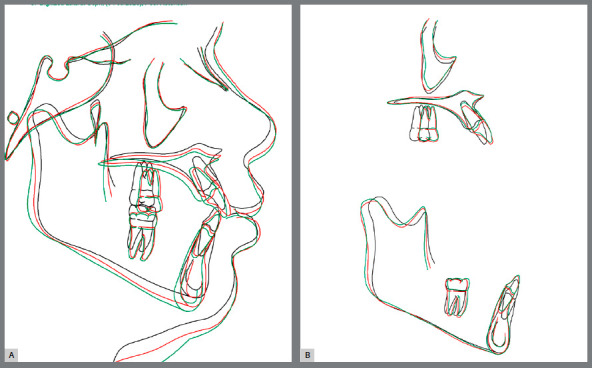
Total (**A**) and partial (**B**) superimpositions of pretreatment (black line), post-treatment (red line), and 26-month retention (green line) cephalometric tracings.

Figure 15 show superimpositions of pretreatment, post-treatment and post-retention cephalometric tracings. The cephalometric tracings superimposition showed a vertical growth and projection of maxillary and mandibular anterior teeth, and anchorage loss in the maxillary arch during treatment. In the post-retention phase, tooth stability and a slight vertical growth were observed. These findings show the dental, skeletal, and soft tissue changes achieved as the result of treatment, and stability in the retention period.

## DISCUSSION

In orthodontic treatment associated with dental trauma, the risk of side effects - such as ankylosis, root resorption, pulp necrosis, root exposure and attachment loss^5^ ^,^ ^10^ ^,^ ^12^ ^-^ ^14^ ^,^ ^23^ ^,^ ^24^ - during orthodontic movement makes the prognosis uncertain, as there is no defined protocol for the evaluation of traumatized and impacted teeth^5^. 

In cases of dental impactions caused by dental trauma, several authors^3,7,8,11,14-17^ have opted for orthodontic traction, even though it may require a longer treatment time and despite its many disadvantages. According to Kuvvetli et al.,^27^ if the condition is diagnosed early, if the stage of root development and crown shape are appropriate, and if the patient complies with the long and difficult procedures involved, the forced tooth eruption can be accepted as the best option for these cases. In fact, Cheng et al^7^, who prospectively and longitudinally evaluated nine cases of children with impacted central incisors using CBCT before treatment, after treatment and one year out of retention, found that traction of the mixed dentition could promote root development and alveolar bone remodeling when the patient is in an early stage of root development, with open apex. The authors also found that the alveolar bone condition of vertically impacted teeth was better than that of horizontally impacted teeth.^3^ ^,^ ^7^ ^,^ ^8^ ^,^ ^11^ ^,^ ^14^ ^-^ ^17^

The available literature also shows that suitable treatment timing is critical for treating malpositioned impacted maxillary central incisors.^7^ ^,^ ^27^ ^,^ ^20^ In the clinical case presented, however, this 13-year-old patient, in addition to having an impacted maxillary central incisor positioned inverted, had a history of TDI, maxillary lateral incisor ankylosis and loss of alveolar bone in the region, raising doubts about prognosis of the traction. The prognosis for traction of a tooth in Nolla’s stage 10 and in an inverted position is unfavorable, considering the root length, the risk of root resorption and the alveolar bone loss on the labial side.^20^

Besides, due to the great lack of space for tooth eruption, the maxillary canines were also impacted, and an incomplete transposition between the maxillary left lateral incisor and maxillary left canine would make canine traction to the lateral incisor region a good option, which could significantly improve the alveolar bone in the area,^28^ since the periodontal ligament and alveolar bone are a functional unit and undergo robust remodeling, leading to osteogenesis in adequate orthodontic tooth movement.^29^ Likewise, the movement of the maxillary right central incisor, replacing the impacted left central incisor, would also stimulate the alveolar bone^28^. Conversely, the option of central incisor traction could fail and increase the risk of alveolar loss.^10^ ^,^ ^12^ ^-^ ^14^ ^,^ ^23^ ^,^ ^24^

The rationale of the decision-making process that culminated in the extraction of the left maxillary central incisor considered the psychological condition of the child and the family in the face of a traumatic case of bullying - which is a worldwide problem, with consequences that extend beyond the immediate period during which the acts are carried out.^30^ Chikaodi et al^30^ and Al-Bitar et al^31^ found that the dentofacial feature about which victims were most commonly bullied is having a gap between the teeth/having missing teeth. They also suggest that this and other malocclusions should be considered a public health issue because of their psychosocial effects, advising that governments should consider giving subsidy for the treatment of malocclusion. In addition, the family’s financial condition was also important for decision-making, and the cost of implants and prostheses would be impracticable, according to the patient’s parents. 

Taking this information into account, it was decided to perform a treatment that would solve the problem that impacted her smile and speech during her adolescence, with less risk of additional bone loss in the maxillary anterior region. Treatment options in cases in which the treatment prognosis is uncertain and present potential risks should be widely discussed with the family, especially in cases of bullying with significant emotional distress.^32^ The challenges related to uncertainty of outcomes and limited purchasing power of the family, and specially the time-consuming nature of the treatment and the fear of having additional complications in tooth with positive prognosis, such as the canines, directly influenced the family decision.

In this treatment option, unlike with implants, there are no risks of increasing the degree of infraocclusion during and after completion of growth, or significant marginal bone loss at tooth surfaces adjacent to the implant, as described by Thilander et al.^33^

Thus, the additional challenge of this treatment was the movement of a right central incisor to the left central incisor location, closing the large gap that resulted from losing one central incisor and one lateral incisor in the same quadrant, which required bilateral mesial movement of teeth. A small number of case reports have shown that incisor movement to the contralateral side can be performed under careful consideration of its biological limits. Most of these cases were conducted in young patients, who were in the early mixed dentition phase, and treatment was completed at approximately 12 years of age.^34^ ^-^ ^38^ These authors suggested that moving a tooth to the contralateral side of the maxilla could affect the course of the nasopalatine canal and its content, leading to adverse clinical symptoms, especially during the permanent dentition.^34^ ^-^ ^38^ Conversely, Bosio et al^22^ performed this movement across the midline in a 13-year-old boy, also in the permanent dentition and, after treatment, CBCT showed that tooth movement had little or no effect on the position of the incisive foramen. In addition, axial views on CBCT showed that the spatial course of the nasopalatine canal was relatively unchanged. Therefore, it appeared that the orthodontic correction included the teeth, periodontal tissues and related attached buccal mucosa, but did not affect the processes of the hard palate^22^, which is similar to the clinical results achieved in the present case.

The present case corroborated the impossibility of moving teeth across the suture.^22^ ^,^ ^34^ ^-^ ^37^ The failure to cross the suture resulted in substantial deviations of the incisive papilla and labial frenum, which came to lie between the maxillary central incisor and left canine - i.e. there was rotation of the anterior portion of the midpalatal suture around the incisive foramen. This movement of the incisive papilla and the upper labial frenum with the relocated tooth was similar to the movement demonstrated in earlier studies^22^ ^,^ ^41^ ^-^ ^43^ on gingival movement. McCollum^37^ stated that this stretched frenum and gingival tissue appear to play little or no role in the relapse of these incisors, as in this case they remained stable for nearly 9 years after retention - similar to the results found in the present case after more than 2 years of retention. 

On the other hand, case reports and studies have shown that if the suture is mineralized, the tooth moves normally.^22^ ^,^ ^34^ ^-^ ^37^ If the suture is not mineralized, however, the midpalatal suture undergoes distortion in the same direction as that of tooth movement; moreover, the connective tissue of the suture is incorporated into the periodontal ligament,^22^ ^,^ ^41^ ^-^ ^43^ which appears as a radiolucent image distal to the central incisor, exactly as observed in the present study. CBCT showed that the right central incisor was successfully moved to the left, inducing deviation of the midpalatal suture to the same side.

Similar results were found in other studies^22^ ^,^ ^41^ ^,^ ^42^ ^,^ ^43^. Bulut and Pasaoglu^43^ termed the current condition as a “slippage phenomenon”. 

However, hygiene of the proximal surface of the maxillary right central incisor was a limitation in this case, due to its inclination to the left side. Before the removal of the orthodontic appliance, placement of a bone graft was indicated to allow the verticalization of the root and the elimination of the defect. However, the family was already satisfied with the results and requested the removal of the appliance as soon as possible, despite the possible periodontal risks. The presence of fixed lingual retainer is a factor that makes cleaning even more difficult, so the patient was emphatically instructed about the need to use dental floss, and is being followed up with biannual periodontal control visits.

The treatment of TDIs is often complex and requires interdisciplinary approach, and should be directed to avoid sequelae.^18^ Then, the therapy of an impacted maxillary central incisor and an ankylosed lateral incisor in the same quadrant due to trauma requires a multidisciplinary approach: Orthodontics, Surgery, Periodontics, Prosthetics and Esthetic Dentistry are essential for successful treatment.^44^ The present patient reported satisfactory dental function, absence of muscle pain or joint alterations, satisfaction with dental and facial aesthetics, and improved quality of life. No significant root resorption was observed as a consequence of the major tooth movement. Moreover, we believe that the long-term stability of results observed in the present case likely had a strong influence on the correction of tongue function and position.

## CONCLUSION

In orthodontic treatment associated with TDIs, the risk of side effects during orthodontic movement should be taken into account. Considering the many risks and consequences involved, a multidisciplinary approach is essential when a central incisor is orthodontically moved across the midline. The final results showed stability of an atypical occlusion, with a with Class II molar relationship and right central incisor replacing the left central incisor, with favorable dental and facial aesthetics. 

## References

[1] Caprioglio C, Olivi G, Genovese MD, Vitale MC (2017). Paediatric laser dentistry Part 3: Dental trauma. Eur J Paediatr Dent.

[2] Diab M, elBadrawy HE (2000). Intrusion injuries of primary incisors Part III: Effects on the permanent successors. Quintessence Int.

[3] Paoloni V, Pavoni C, Mucedero M, Bollero P, Laganà G, Cozza P (2013). Post-traumatic impaction of maxillary incisors diagnosis and treatment. Ann Stomatol.

[4] Zaror C, Martínez-Zapata MJ, Abarca J, Díaz J, Pardo Y, Pont À (2018). Impact of traumatic dental injuries on quality of life in preschoolers and schoolchildren a systematic review and meta-analysis. Community Dent Oral Epidemiol.

[5] Sarica I, Derindag G, Kurtuldu E, Naralan ME, Caglayan F (2019). A retrospective study do all impacted teeth cause pathology?. Niger J Clin Pract.

[6] Mortazavi H, Baharvand M (2016). Jaw lesions associated with impacted tooth a radiographic diagnostic guide. Imaging Sci Dent.

[7] Cheng C, Li X, Liu H (2016). Evaluation of the orthodontic treatment outcome in patients with impacted maxillary central incisor in the mixed dentition. Zhonghua Kou Qiang Yi Xue Za Zhi.

[8] Shi XR, Hu Z, Wang XZ, Sun XY, Zhang CY, Si Y (2015). Evaluation of the effect of the closed-eruption technique on impacted immature maxillary incisors. Chin J Dent Res.

[9] Lygidakis NN, Chatzidimitriou K, Theologie-Lygidakis N, Lygidakis NA (2015). Evaluation of a treatment protocol for unerupted maxillary central incisors: retrospective clinical study of 46 children. Eur Arch Paediatr Dent.

[10] Ling KK, Ho CT, Kravchuk O, Olive RJ (2007). Comparison of surgical and non-surgical methods of treating palatally impacted canines II. Aesthetic outcomes. Aust Orthod J.

[11] Gebert TJ, Palma VC, Borges AH, Volpato LE (2014). Dental transposition of canine and lateral incisor and impacted central incisor treatment a case report. Dental Press J Orthod.

[12] Campbell KM, Casas MJ, Kenny DJ (2005). Ankylosis of traumatized permanent incisors pathogenesis and current approaches to diagnosis and management. J Can Dent Assoc.

[13] Campbell KM, Casas MJ, Kenny DJ (2007). Development of ankylosis in permanent incisors following delayed replantation and severe intrusion. Dent Traumatol.

[14] Frank CA, Long M (2002). Periodontal concerns associated with the orthodontic treatment of impacted teeth. Am J Orthod Dentofacial Orthop.

[15] de Oliveira Ruellas AC, Mattos CT (2012). Multidisciplinary approach to a traumatized unerupted dilacerated maxillary central incisor. Angle Orthod.

[16] Farronato G, Maspero C, Farronato D (2009). Orthodontic movement of a dilacerated maxillary incisor in mixed dentition treatment. Dent Traumatol.

[17] Kocadereli I, Turgut MD (2005). Surgical and orthodontic treatment of an impacted permanent incisor case report. Dent Traumatol.

[18] Oliveira Werlich M, Honnef LR, Silva Bett JV, Domingos FL, Pauletto P, Dulcineia Mendes de Souza B (2020). Prevalence of dentofacial injuries in contact sports players A systematic review and meta-analysis. Dent Traumatol.

[19] Smailiene D, Sidlauskas A, Bucinskiene J (2006). Impaction of the central maxillary incisor associated with supernumerary teeth initial position and spontaneous eruption timing. Stomatologija.

[20] Sun H, Hu R, Ren M, Lin Y, Wang X, Sun C (2016). The treatment timing of labial inversely impacted maxillary central incisors a prospective study. Angle Orthod.

[21] Mendes RA, Rocha G (2004). Mandibular third molar autotransplantation literature review with clinical cases. J Can Dent Assoc.

[22] Bosio JA, Bradley TG, Hefti AF (2011). Moving an incisor across the midline a treatment alternative in an adolescent patient. Am J Orthod Dentofacial Orthop.

[23] Topouzelis N, Tsaousoglou P, Pisoka V, Zouloumis L (2010). Dilaceration of maxillary central incisor a literature review. Dent Traumatol.

[24] Malmgren O, Goldson L, Hill C, Orwin A, Petrini L, Lundberg M (1982). Root resorption after orthodontic treatment of traumatized teeth. Am J Orthod.

[25] Consolaro CA, Consolaro RB, Francischone LA (2010). Orthodontic forced eruption possible effects on maxillary canines and adjacent teeth: part 3: dentoalveolar ankylosis, replacement resorption, calcific metamorphosis of the pulp and aseptic pulp necrosis. Dental Press J Orthod.

[26] Burstone CJ (2001). Biomechanics of deep bite correction. Semin Orthod.

[27] Kuvvetli SS, Seymen F, Gencay K (2007). Management of an unerupted dilacerated maxillary central incisor a case report. Dent Traumatol.

[28] Gkantidis N, Christou P, Topouzelis N (2010). The orthodontic-periodontic interrelationship in integrated treatment challenges a systematic review. J Oral Rehabil.

[29] Baloul SS (2016). Osteoclastogenesis and osteogenesis during tooth movement. Front Oral Biol.

[30] Chikaodi O, Abdulmanan Y, Emmanuel AT, Muhammad J, Mohammed MA, Izegboya A (2017). Bullying, its effects on attitude towards class attendance and the contribution of physical and dentofacial features among adolescents in Northern Nigeria. Int J Adolesc Med Health.

[31] Al-Bitar ZB, Al-Omari IK, Sonbol HN, Al-Ahmad HT, Cunningham SJ (2013). Bullying among Jordanian schoolchildren, its effects on school performance, and the contribution of general physical and dentofacial features. Am J Orthod Dentofacial Orthop.

[32] Kazanci F, Aydogan C, Alkan Ö (2016). Patients' and parents' concerns and decisions about orthodontic treatment. Korean J Orthod.

[33] Thilander B, Odman J, Lekholm U (2001). Orthodontic aspects of the use of oral implants in adolescents a 10-year follow-up study. Eur J Orthod.

[34] Cookson AM (1981). Movement of an upper central incisor across the midline. Br J Orthod.

[35] Follin ME (1985). Orthodontic movement of maxillary incisor into the midline A case report. Swed Dent J.

[36] Melnik AK (1993). Orthodontic movement of a supplemental maxillary incisor through the midpalatal suture area. Am J Orthod Dentofacial Orthop.

[37] McCollum AG (1999). Crossing the midline a long-term case report. Am J Orthod Dentofacial Orthop.

[38] Teufelberger W, Schachner P, Schicher-Kucher N, Bantleon HP (2008). Long-term results of shifting a central incisor across the midline case report. Inf Orthod Kieferorthop.

[39] Spear FM, Kokich VG (2007). A multidisciplinary approach to esthetic dentistry. Dent Clin North Am.

[40] Rosa M, Zachrisson BU (2007). Integrating space closure and esthetic dentistry in patients with missing maxillary lateral incisors. J Clin Orthod.

[41] Garib DG, Janson G, dos Santos PB, de Oliveira Baldo T, de Oliveira GU, Ishikiriama SK (2012). Orthodontic movement of a maxillary incisor through the midpalatal suture a case report. Angle Orthod.

[42] Pair J (2011). Movement of a maxillary central incisor across the midline. Angle Orthod.

[43] Bulut H, Pasaoglu A (2017). Multidisciplinary management of a fused maxillary central incisor moved through the midpalatal suture a case report. Korean J Orthod.

[44] Liu F, Wu TT, Lei G, Fadlelseed AFA, Xie N, Wang DY (2020). Worldwide tendency and perspectives in traumatic dental injuries A bibliometric analysis over two decades (1999-2018). Dent Traumatol.

